# Self-assembly modified-mushroom nanocomposite for rapid removal of hexavalent chromium from aqueous solution with bubbling fluidized bed

**DOI:** 10.1038/srep26201

**Published:** 2016-05-18

**Authors:** Fei Xu, Xu Liu, Yijiao Chen, Ke Zhang, Heng Xu

**Affiliations:** 1 Key Laboratory of Bio-resources and Eco-environment (Ministry of Education), College of Life Science, Sichuan University, Chengdu, Sichuan 610064, China

## Abstract

A self-assembled modified *Pleurotus Cornucopiae* material (SMPM) combined with improved Intermittent Bubbling Fluidized Bed (IBFB) was investigated to remove the hexavalent chromium ions in aqueous solution. After the modification, the powder-like raw material gradually self-assembled together to SMPM, which had crinkly porous structure, improved the Cr-accommodation ability in a sound manner. Optimized by Taguchi method, Cr(VI) removal efficiency was up to 75.91% and 48.01% for 100 mg/L and 500 mg/L initial concentration of Cr(VI), respectively. Results indicated that the metal removal was dependent on dosage of adsorbent, particle diameter and treatment time. The experimental data obtained from the biosorption process was successfully correlated with Freundlich isotherm model. Thermodynamic study indicated the endothermic nature of the process. The results confirmed that self-assembly modified *Pleurotus Cornucopiae* material could be applied for the removal of heavy metal from wastewater in continuous fluidized bed process.

To date, the heavy metal pollutants have become a great issue in living environment. Those pollutants can be generated by a variety of industries such as electroplating, leather tanner, textile dyeing, steel fabrication and wood preservation treatment produce. As one of the toxic heavy metal pollutants^1^, chromium has two most stable states in aquatic environment: Cr(III) and Cr(VI). But Cr(VI) is 500 times more toxic to living organisms than Cr(III). Meanwhile, Cr(VI) can be easily accumulated in the food chain. Thus, this highly toxic metal pollutant can cause severe health problems in human beings, such as simple skin irritation, critical lung carcinoma and so on^2^,^3^. The World Health Organization (WHO) recommended maximum allowable limit for Cr(VI) in drinking water as 0.05 mg/L^4^.

To comply with legal requirements and preserve the drinking water quality, it is essential to lower down the concentration of Cr(VI) to the accepted level before it being discharged into the environment. Thus far, several conventional methods have been carried out to remove Cr(VI) from aqueous solution, including chemical precipitation, ion exchange, adsorption, membrane filtration and electrochemical treatment, etc^5^. Among these technologies, physical adsorption has been widely investigated due to its high efficiency, low-cost, high regeneration and reuse ability^6^. Various materials have been employed as an adsorbent for removal of Cr(VI) ions, including activated carbon (AC)^7^, carbon nanotubes (CNTs)^8^, lignite^9^ and so on.

Biomaterials have also been widely investigated as adsorbent for removing Cr(VI) ions from aqueous solution due to its non-toxicity, biocompatibility, biodegradability and low-cost nature^5^. Although biosorbent possesses a lot of advantages, it is still restricted to disadvantages related to small size, low density and poor mechanism strength, thus limiting its application in the heavy metal removal^5^,^10^. Therefore, various biomass immobilization techniques have been carried out to solve these problems. However, none of them were convenient or economic enough to be applied in real situation of Cr(VI) seperation^10^. Moreover, the biomass immobilization would shield the interface between pollutant and biosorbent. Therefore, a new approach is required to improve the properties of biosorbent.

In the present study, a self-assembly modified *Pleurotus cornucopiae* material (SMPM) is employed as an adsorbent for the removal of Cr(VI) from polluted wastewater and is reported for the first time. An improved Intermittent Bubbling Fluidized Bed (IBFB) was applied as reactor. The Taguchi method was performed to study the effect of various parameters such as dosage, treatment time, concentration of modifier and particle size. At the same time, the optimum operation conditions under the influence of external interference (such as initial concentration, pH and temperature) were also investigated. Langmuir and Freundlich models were used to explore the thermodynamic nature of the adsorption process. In addition, SMPM was characterized using Scanning Electron Microscopy (SEM), Energy Dispersive X-ray analysis (EDX) and Fourier Transform Infrared (FTIR) techniques.

## Materials and Methods

### Adsorbent preparation and modification

*Pleurotus cornucopiae* was purchased from market nearby the campus in Chengdu, Sichuan Province, China. Washed by double distilled deionized water (ddH_2_O) and dried at 50 °C for 3 d by using air-blower-driven drying closet. Then, grounded using a pulverizing mill (JoYoung, JYL-350B) and sieved through three kinds of screen: 40-mesh, 60-mesh and 100-mesh, the obtained particle sizes were: 0.30 mm < d < 0.45 mm, 0.15 mm < d < 0.30 mm and d < 0.15 mm, respectively.

For surface modification, 30 g uniformed biomass particles were agitated in 500 ml of the dodecyl dimethyl benzyl ammonium bromide solution. After 24 h, filtered and washed with ddH_2_O until the bromide ion had been eliminated. Finally, placed in drying closet at 30 °C for 24 h for self-assembling.

### Chemicals and equipment

All the chemicals and reagents utilized in the study were analytical grade (Kelong Chemical Reagent Factory, Chengdu, China). Potassium dichromate was used as adsorbate. The stock adsorbate solutions (1000 mg/L and 5000 mg/L) were prepared by dissolving 2.828 g and 14.140 g of potassium dichromate in 1 L of ddH_2_O, respectively. All working solutions were obtained by dilution. Cr(VI) concentration was determined by flame atomic adsorption spectrometry (VARIAN, SpectrAA-220Fs).

### Experimental method

The Taguchi method, which was established by Genichi Taguchi in 1980s^11^, has been widely used as a systematic approach to optimize the design parameters under the given conditions^12^,^13^,^14^. This approach can significantly minimize the overall testing time and the experimental costs^12^,^13^,^14^,^15^,^16^. By using the specially designed orthogonal array, the optimum experimental conditions can be determined^13^.

**Figure 1:**
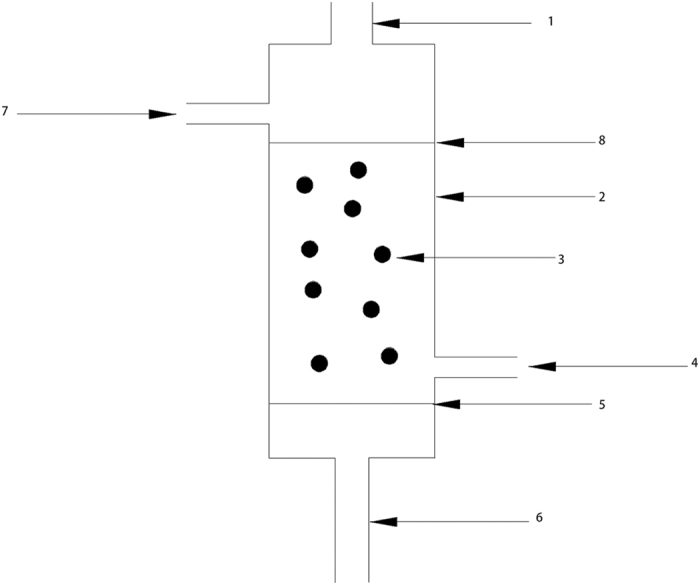
Diagram of Intermittent Bubbling Fluidized Bed (IBFB). 1- adsorbent inlet, 2- reactor, 3- adsorbent, 4- effluent, 5-gas distribution plate and adsorbent barrier, 6- air inlet, 7- treatment solution input, 8- separator.

Accordingly, an analysis of the signal-to-noise (S/N) ratio is required to evaluate the experimental results. Commonly, three types of S/N ratio analysis are applicable: (1) lower is better (LB), (2) nominal is best (NB), (3) higher is best (HB). Because of the purpose of this study is to acquire the optimized operational conditions with maximum Cr(VI) removal ratio, the S/N ratio with HB characteristics is required, which can be described by equation (1):


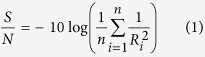


where n is the number of experiments under the same conditions, R represents the Cr(VI) removal efficiency, which can be calculated using equation (2):


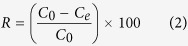


where C_0_ and C_e_ represent the initial and equilibrium concentrations of Cr(VI) (mg/L), respectively. The experiment was conducted in a laboratory scale IBFB, which was made of Plexiglas with an inner diameter of 0.04 m and height of 0.6 m (Fig. 1).

### Characterization of SMPM

Scanning Electron Microscopy (SEM) (JSM-5900LV, Japan) was used to identify the surface morphology features of raw *Pleurotus cornucopiae* material (RPM) and SMPM. The chemical elements on the surface of biomass and the main functional groups were analyzed by Energy Dispersive X-ray analysis (EDX) and Fourier Transform Infrared (FTIR) spectrometer (NEXUS-650, America).

### Optimization study

In this study, four controllable factors were considered and three factors were considered as noise, each controllable factor had three levels and each noise factor had two levels (Table 1). Hence, an L_9_ orthogonal array was chosen to build the inner array and an L_4_ orthogonal array was chosen to build the outer array. By using JMP 10 (SAS, USA), a Taguchi method array was automatically created. A series of aqueous of Cr (VI) with 500 ml volume was treated within IBFB. The treatment time were 2 to 6 hours, the dosage of adsorbent were 0.5 to 5.0 g, diameters of adsorbent were 0.15 to 0.45 mm and the biomass was modified by series different concentrations of modifier (0.5~2%). The initial concentration of Cr(VI) solution, pH and temperature were chosen as noise factors with two levels: 100 mg/L and 500 mg/L; pH = 2 and pH = 5; 20 °C and 30 °C, respectively.

The analysis of mean (ANOM) statistical approach was conducted to evaluate the optimal conditions under the influence of noises. Initially, the mean of the S/N ratio of each controllable factor at a certain level should be calculated by the following equation:


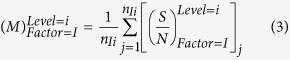


In equation (3), *n*_*Ii*_ represents the number of appearances of factor I in the level I under the influence of four combinations of three noises, 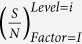 represents the S/N ratio of factor I in level i, j represents the appearance sequence of prior variable in Table 2.

According to the above equation, the mean of the S/N ratio of other factors in a certain level can be determined, as well as the optimal conditions. Eventually, the confirmation experiment was carried out to verify the results.

In addition, the analysis of variance (ANOVA)^13^ was also used to analyze the influence of each controllable factor on the removal of Cr(VI). The contribution rate of each factor, *ρ*, is given by equation (4):


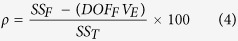


where *DOF*_*F*_ represents the degree of freedom for each factor, which is obtained by subtracting one from the number of the level of each factor (L). The total sum of squares, *SS*_*T*_, is given by


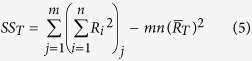


where 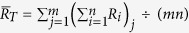, m represents the number of experiments carried out in this study, and n represents the number of repetitions. The factorial sum of squares, *SS*_*F*_, is given by


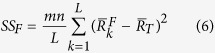


where 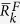 is the average value of the removal efficiency of a certain factor in the *k*th level. And the variance of error, *V*_*E*_, is given by


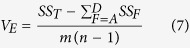


### Sorption isotherms study

In order to study the sorption isotherms, a series of chromium solutions with different initial concentrations were prepared (100, 200, 300 and 500 mg/L, respectively). The experimental conditions were as follows: the dose of the SMPM was 5 g, which was modified by 1% (v/v) of dodecyl dimethyl benzyl ammonium bromide solution for 24 h; the average particle diameter of the SMPM was 0.3 mm < d < 0.45 mm, and the adsorption process was lasted for 2 h. When sorption reached equilibrium, q (mg/g), the weight of chromium adsorbed per unit of dry adsorbent weight can be calculated by:


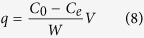


where *C*_0_ and *C*_*e*_ (mg/g) are the initial and equilibrium concentrations of Cr respectively. *V* (L) is the volume of the solution, *W* (g) is the mass of dry adsorbent which was used^13^. Langmuir and Freundlich isotherm models were used to analyze the sorption equilibrium data of the adsorption process^14^. The Langmuir model is given by:


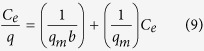


where *q* (mg/g) is the weight of chromium adsorbed per unit of dry adsorbent weight at equilibrium, *C*_*e*_ (mg/L) is the equilibrium concentration of metal ions in liquid phase. *q*_*m*_ (mg/g) is the maximum monolayer capacity, *b* (l/mg)is the adsorption affinity onto the adsorption sites and it is related to energy of adsorption, both of them are Langmuir constants, which can be calculated through the slope and intercept of the linear plot with 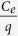 versus *C*_*e*_.

The Freundlich model is an empirical equation based on heterogeneous surfaces. Compare to the Langmuir model, the Freundlich model suggesting the binding sites are not equivalent and independent^17^,^18^. The Freundlich model is given by:


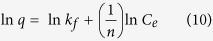


where *q* (mg/g) is the weight of chromium adsorbed per unit of dry adsorbent weight at equilibrium, *C*_*e*_ (mg/l) is the equilibrium concentration of metal ions in liquid phase. *k*_*f*_ (mg/g) represents the sorption capacity and *n* (l/mg) represents the sorption intensity. *k*_*f*_, the Freundlich constant can be calculated through the slope and intercept of the linear plot with ln *q* versus ln *C*_*e*_.

## Results and Discussion

### Optimization study

#### Optimum conditions

According to the Taguchi method, Test 1–9 were accomplished. The Cr (VI) removal efficiency for each test was calculated by equation (2). Using equation (1), the S/N ratio of each test condition can be determined. The highlights in Table 2 represent the maximum value of S/N ratio among all nigh tests. The mean of the S/N ratio of each controllable factor at a certain level, 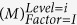, was given by equation (3), which referred to the effect of each level of each factor to the response. In Table 3, the maximum value of 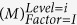 among all four factors in three levels was bolded. These maximum values indicated the optimization conditions for Cr (VI) removal process^13^,^14^,^19^. The optimum conditions are as follows: the dosage of adsorbent is 5 g; duration of treatment is 2 hours; the concentration of modifier is 1% (v/v) and the diameter of adsorbent is between 0.3 mm and 0.45 mm.

#### Dosage effect of SMPM

**Figure 2:**
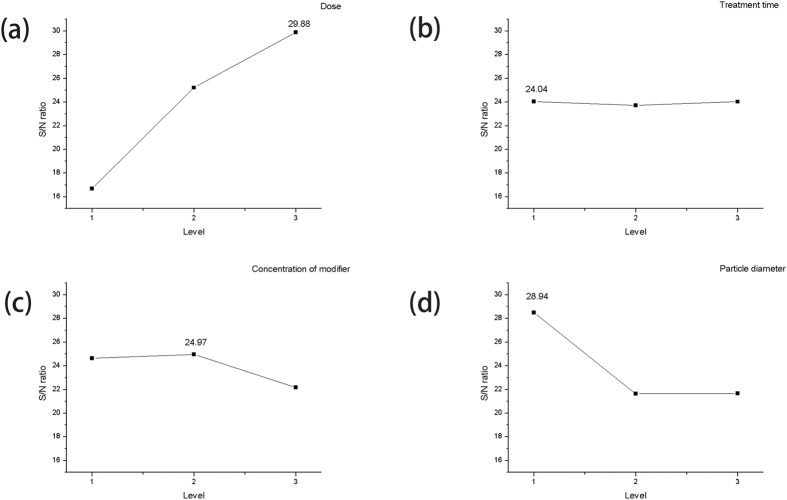
The effect of all controllable factors on S/N ratio in the removal of Cr(VI).

Figure 2a shows the effect of dosage of SMPM on S/N ratio in the removal of Cr(VI). According to the data, the S/N ratio reached the peak, 29.88, when the dosage of SMPM was 5 g. While lowest S/N ratio emerged when the dosage was 0.5 g, which was only 16.69. This result suggested that, the more adsorbents participant, the more pollutants can be removed. This was because the increasing dosage of adsorbent led to the increasing adsorption sites^1^,^3^,^20^,^21^.

#### Effect of treatment time

**Figure 3:**
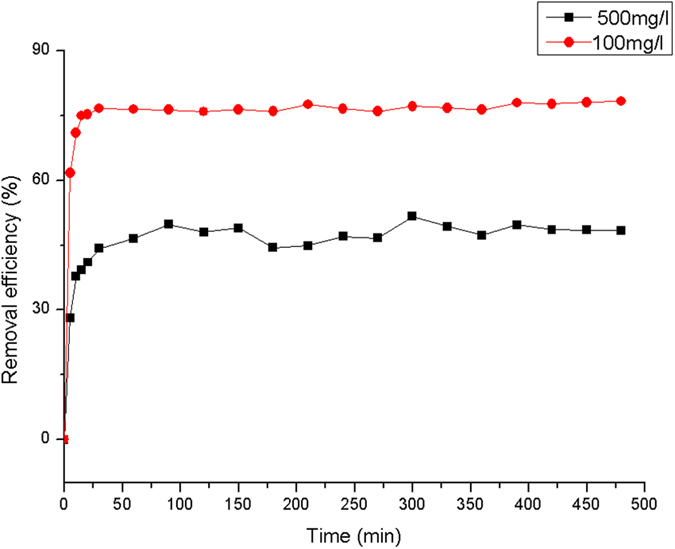
The verification tests running under optimum conditions, at room temperature. The initial concentrations of Cr(VI) were 100 mg/l and 500 mg/l, the pH were 3.96 and 3.57, respectively.

According to Fig. 2b, three levels of treatment time led to slightly differences on S/N ratio. The highest S/N ratio value was 24.04, when treatment time was 2 h while the lowest value was 23.72, when treatment time was 6 h. This result indicated that the time for the process to reach the equilibrium state could be less than 2 h. In order to verify this standpoint, two verification tests were performed. These tests were carried out according to the aforementioned optimum conditions: the temperature was 18 °C, the initial concentrations of solution were 100 and 500 mg/L and the pH of solution were 3.96 and 3.57, respectively. Figure 3 shows the results of the verification tests. During the verification tests, the removal efficiency increased rapidly in the first 10 min. After that, the difference had shown up between 100 and 500 mg/L solution. When the concentration of solution was 100 mg/L, the removal efficiency rapidly increased to 71% in the first 10 min. Then, the removal efficiency kept at a stable level of 76–78%. However, the removal efficiency slightly increased after 10 min and reached a stable level until 100 min in the high-concentration solution. Therefore, the optimum treatment time should be 120 min (2 h) for both 100 mg/L and 500 mg/L Cr(VI) solutions.

#### Concentration effect of modifier

As shown in Fig. 2c, when the concentration of modifier was 1%, the S/N ratio was the highest, 24.97. When the concentration of modifier decreased to 0.5% or increased to 2%, the S/N ratio decreased to 24.65 and 22.17, respectively. These results are similar to the Jing’s research^3^, which had reported that when the concentration of modifier was below a certain level the removal efficiency increased, but decreased when the concentration was higher than that level. According to his research, the enhanced electrostatic interaction between positively charged head group of cationic modifier might caused the increase^3^. Furthermore, the loosely bound amine released from admicelles and the competition of anion between cationic modifier bounding to the surface of biomass and that in solution might also play an important role^22^.

#### Effect of the particle diameter

As shown in Fig. 2d, the S/N ratio reached the highest level, 28.94, when the diameter was between 0.3 and 0.45 mm. However, the S/N ratio decreased while the diameter reduced. SEM results indicated that the surface became more smoothness when diameter was below a certain level. The vanishing of drapes and polyporous structure significantly decreased the superficial area of the material, which would lead to the deceasing of sorption ability.

#### Noise factors

**Figure 4:**
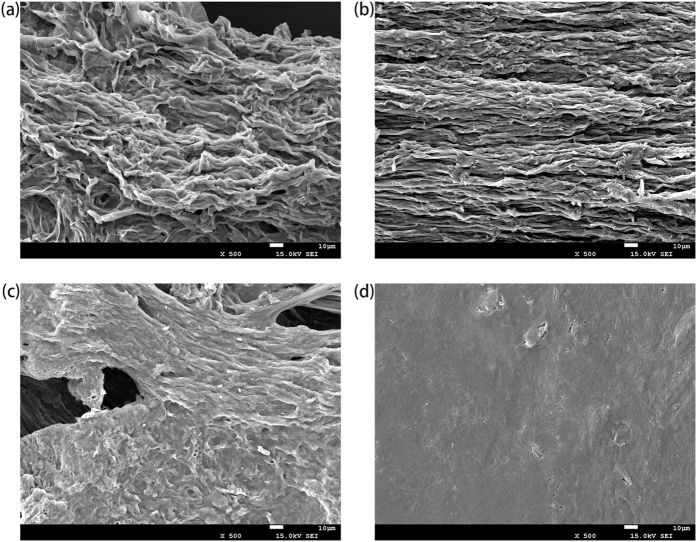
(**a**)SEM images of RSMPM, (**b**) SMPM assembled by particles with diameter at 0.30 mm < d < 0.45 mm, (**c**) SMPM assembled by particles with diameter at 0.15 mm < d < 0.30 mm and (**d**) SMPM assembled by particles with diameter at d < 0.15 mm.

Many researches had confirmed that pH, initial concentration and temperature of adsorbate solution had significant impacts on the absorption efficiency. Previous studies showed that in the pH range of 2 to 6, Cr(VI) removal decreased while the pH increased^1^,^17^,^20^,^23^. Those studies indicated that the maximum adsorptions were obtained at pH 2. It is mainly because the pH of the solution changes the form of the chromium ion, protonation level and the surface charge of the adsorbent^3^,^5^,^24^. At pH 2, the major form of chromium ion is Cr_2_O_7_^2−^. However, as the pH increases the concentration of Cr_2_O_7_^2−^ decreases and the concentration of CrO_4_^2−^ increases. Meanwhile, as the pH increases, the surface charge of adsorbent become more negative, which reduces the electrostatic attraction towards negatively charged chromium ion. Similarly, the concentration of hydroxide ion increases as the pH increases, which could double the competition of chromium ion and hydroxide ion to be adsorbed on the surface of adsorbent^1^,^3^,^10^,^17^. The increasing initial concentration would trigger the decreasing of the removal efficiency. Singh *et al.*^25^ had reported that as the initial concentration increased from 10 mg/L to 50 mg/L, the removal efficiency of Cu(II) and Pb(II) decreased from 97% to 59.6%, 99.2% to 80.2%, respectively. In this study, the efficiency of Cr(VI) removal had shown the same tendency. According to Fig. 4, under the same experiment conditions (the dosage of adsorbent was 5 g; duration of treatment was 2 hours; the adsorbent was modified in 1% (v/v) dodecyl dimethyl benzyl ammonium bromide solution for 24 h and the diameter of adsorbent was between 0.3 mm and 0.45 mm), the removal efficiency decreased from 75.91% to 48.01%, while the initial concentration of Cr(VI) increased from 100 to 500 mg/L. This was caused by the available adsorption sites limitation and this limitation is also being considered as the direct result of the corresponding of removal efficiency decreasing^26^,^27^,^28^. Obviously, the total available adsorption sites of certain adsorbent dosage are limited. Thus, when the adsorbate concentration increases, the ratio of ions that could bond to the sites decreases. Most adsorption experiments preformed at room temperature^1^,^23^,^28^,^29^ and it had been demonstrated that temperature (from 20 to 35 °C) had less effect on the adsorption process^14^,^30^. Meanwhile, due to the endothermic nature of the process, the higher temperature reinforces the removal ability by increasing the adsorbent’s surface activity and kinetic energy^24^. Therefore, temperature has a minimal impact on biosorption compare to other factors.

The three factors mentioned above can affect the removal outcome in the lab-scale experiments. Nonetheless, they are too difficult to control in the practical industrial applications due to the cost and other reasons. For instance, hydrochloric acid, which can be used to adjust the pH, is strictly controlled according to the national security policy. Therefore, controlling those three factors in an optimum range during industrial-scale operation could significantly increase the operating cost and infrastructure construction difficulties. Thus, in this study the pH, initial concentration and temperature of adsorbate solution were considered as noise factors.

### Contributions of controllable factors

Firstly, the average value of the removal efficiency of a certain factor in the *k*th level, 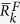, can be calculated from *R*_*i*_ in Table 2. SS_F_, the factorial sum of squares, can be calculated by substituting 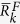 and 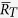 (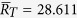) into equation (6), the results are listed in Table 4 and the results of 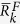 are shown in Table 5. SS_T_, the total sum of squares, was 31420.366. The variance of error, V_E_, was obtained by using equation (7). In the end, substitute all known variables into equation (4), the contribution ratio of each factor can be determined sequentially, and the results are shown in Table 4. It could be concluded from the results that the dosage of adsorbent was the most influential factor on the adsorption process, whose contribution ratio was 29.582%; particle diameter of the adsorbent had second significant influence on the process. The treatment time and concentration of modifier did not have great influence on the adsorption process, and the contribution ratios of them were only 13.79% and 1.34%, respectively.

### Adsorption isotherms

Adsorption isotherm reveals the equilibrium relationship between the adsorbate concentration in the liquid phase and the solid phase at constant temperature, which has significant importance on determining the mechanism of adsorption. Langmuir and Freundlich models were chosen to describe the equilibrium characteristics of the biosorption in this study. These two isotherm models correspond to homogeneous and heterogeneous adsorbent surfaces, respectively^28^,^31^,^32^. The results of adsorption isotherm are listed in Table 6. According to the regression coefficient, R^2^, Freundlich model was more suitable for describing the process of sorption by SMPM in present study, the R^2^ for Freundlich was 0.999, and the R^2^ for Langmuir was 0.981. As an empirical equation, the Freundlich isotherm model is based on heterogeneous surfaces, which suggests that binding sites are not equivalent and/or independent^17^,^18^. Therefore the results of adsorption isotherms indicated that the heterogeneity of Cr(VI) ions to the binding sites which might probably due to the active groups on the SMPM surface, like –OH, -COO- and so on^17^. The parameters of Freundlich isotherm model are shown in Table 6. The sorption intensity (*n*, l/mg) in this study was 1.306 l/mg (1 < *n* < 10), indicating that the adsorption between Cr ions and SMPM is favorable^18^,^33^.

### Thermodynamic study of biosorption

The parameters of thermodynamics associated with the biosorption process such as free energy change (Δ*G*^0^, kJ mol^−1^), standard enthalpy change (Δ*H*^0^, kJ mol^−1^) and standard entropy change (Δ*S*^0^, kJ mol^−1^ K^−1^), can be calculated by the following equations^28^:


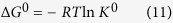



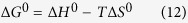


where R is the universal gas constant (8.314 J mol^−1^ K^−1^), T is the absolute temperature (K). The values of Δ*H*^0^ and Δ*S*^0^ can be calculated through the intercept and slop of the linear plot with 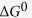 versus T^6^. K^0^ is the equilibrium constant^28^ which is given by:


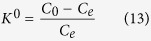


The calculated values of thermodynamic parameters are shown in Table 7. The negative values for Δ*G*^0^ at three different temperatures indicated the thermodynamic feasibility and spontaneous nature of biosorption process. The positive value of Δ*H*^0^ revealed the endothermic nature of the process^20^,^31^,^34^. The magnitude of Δ*H*^0^ obtained in the present work (17.532 kJ mol^−1^) was between 2.1 and 20.9 kJ mol^−1^ representing that physical adsorption might be involved in the adsorption process. While the rang of Δ*H*^0^ was 20.9–418.4 kJ mol^−1^ representing that chemisorption might be involved^20^,^35^. The positive value of Δ*S*^0^ indicated the increased randomness at the solid/liquid interface during Cr sorption^20^.

### Characterization of RPM, SMPM

#### SEM results

SEM images of RPM and SMPM are presented in Fig. 4. Figure 4a reveals that the surface of unmodified material was uneven and polyporous. However, as the diameter of particle decreased the surface of material became smoother. Figure 4b–d clearly indicate that when the diameter was 0.30 mm < d < 0.45 mm, the surface structure was identical to RPM, which suggested the modification can barely change the original structure at this scale of particle diameter. When the particles were smaller than 0.15 mm, the surface of SMPM became flatness and all drapes vanished.

#### EDX results

**Figure 5:**
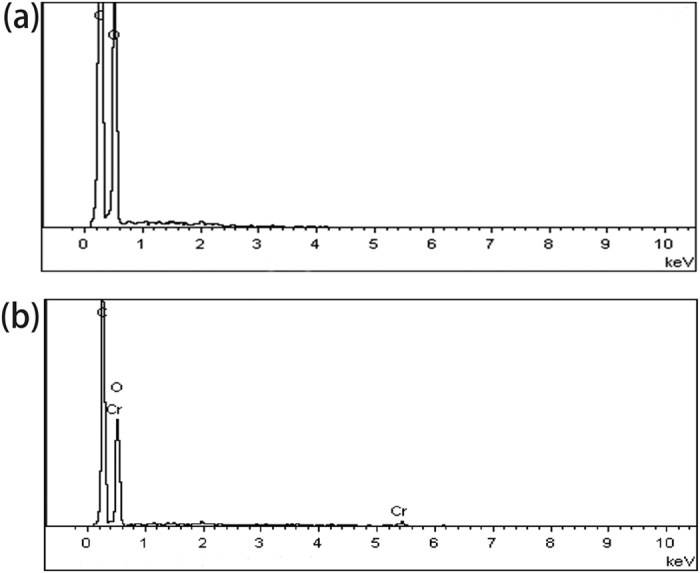
(**a**) EDX spectrum of SMPM and (**b**) SMPM + Cr.

The EDX can help to identify the composition of elements on the surface of the SMPM. As shown in Fig. 5a, the surface of SMPM was composed of carbon and oxygen before the adsorption. The weight percentages of these two elements were 57.85% and 42.15%, respectively. After the adsorption, chromium was identified on the surface of SMPM, and its weight percentage was 0.94%. According to Fig. 5b, the new peaks at 0.6 and 5.4 keV in EDX spectrum of adsorbed SMPM confirmed the chromium adherence onto the surface of SMPM.

#### FTIR analysis

**Figure 6:**
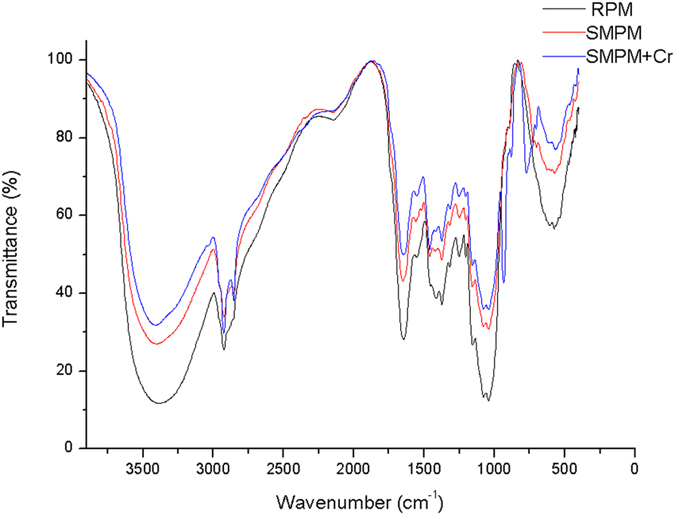
FTIR spectra of raw *Pleurotus cornucopiae* material (RPM), self-assembled modified *Pleurotus cornucopiae* material (SMPM) and after adsorption of Cr on SMPM (SMPM+Cr).

The infrared spectra of RPM, SMPM and SMPM + Cr (after adsorption of Cr on SMPM) were demonstrated in Fig. 6. According to these curves, the broad band observed at about 3400 cm^−1^ indicated the existence of –OH and –NH groups on both unloaded and Cr-loaded biomass. It had been reported that all of the biosorbent have intense absorption bands around 3500 – 3200^36^. The spectra of unloaded and Cr-loaded biomass also displayed absorption peaks at 2924 and 2854 cm^−1^, corresponding to stretching of the C-H bonds of the methyl and methylene groups^36^,^37^. The region between 1650 and 1500 cm^−1^ represented the *C* ≡ *C* stretching in aromatic rings^38^. The peak observed at 1646 and 1552 cm^−1^ for RPM could be attributed to this vibration. This band exhibited a slightly shift to a higher frequency after modification (1652.18 and 1557.94 cm^−1^) as well as adsorption (1651.97 and 1557.63 cm^−1^). The band observed around 1078 cm^−1^ relating to the *C* = *O* bonds confirmed the chitin structure of the SMPM^38^.

Based on the analysis before and after metal sorption, the FTIR results suggested that the chitin structure presented in the biomass, which were more accentuated for Cr sorption. It could also be concluded that on the surface of SMPM, –NH_2_, –OH and –COOH provide the binding site for Cr(VI).

## Conclusions

SMPM has an uneven surface and polyporous structure. FTIR analysis revealed that -NH_2_, -OH and -COOH provided the binding site for Cr(VI). Under the optimum conditions, SMPM can remove Cr(VI) efficiently with the removal efficiency of 75.91%. Among all the controllable factors, the dosage of adsorbent had the largest contribution on the adsorption process, which means that increasing the amount of adsorbent could improve the removal of pollutants significantly. The adsorption in this study fit the Freundlich isotherm model better suggested that on the surface of SMPM, –NH_2_, -OH and -COOH caused a heterogeneity sorption of Cr(VI) to binding site. In conclusion, the biomass system proposed in this study could be an economic, effective and eco-friendly option for the removal of reactive dyes from aqueous media.

## Additional Information

**How to cite this article**: Xu, F. *et al.* Self-assembly modified-mushroom nanocomposite for rapid removal of hexavalent chromium from aqueous solution with bubbling fluidized bed. *Sci. Rep.* **6**, 26201; doi: 10.1038/srep26201 (2016).

## Figures and Tables

**Table 1:** Controllable and noise factors of Taguchi method.

			Level		
Factor		Description	1	2	3
Controllable factors	A	Dose (g)	0.5	1	5
	B	Treatment time (h)	2	4	6
	C	Concentration of modifier (%)	0.5	1	2
	D	Particle diameter (mm)	0.3 < d < 0.45	0.15 < d < 0.3	d < 0.15
Noise Factors	1	Concentration of adsorbate (mg/L)	100	500	
	2	pH of the adsorbate	2	5	
	3	Temperature (°C)	10	30	

**Table 2:** The result of the Taguchi method.

Tests					Noises factors								S/N
	Factors				− − +		− + −		+ − −		+ + +		
					A	B	C	D	R%							
1	A1	B1	C1	D1	5.72	11.89	33.04	32.58	22.32	25.19	9.02	11.84	22.08
2	A1	B2	C2	D2	1.44	5.17	31.79	26.89	10.91	13.14	7.92	6.19	15.24
3	A1	B3	C3	D3	7.42	13.45	27.48	21.71	18.02	12.82	1.3	3.21	12.76
4	A2	B1	C2	D3	18.8	15.4	39.3	38.09	10.02	28.97	8.64	12.6	24.09
5	A2	B2	C3	D1	16.95	19.15	63.16	59.74	43.78	41.11	15.14	23.15	27.81
6	A2	B3	C1	D2	9.51	11.77	49.32	45.66	33.91	28.07	12.43	12.28	23.75
7	A3	B1	C3	D2	19.75	24.64	27.65	28.59	55.04	52.21	7.69	17.03	25.94
8	A3	B2	C1	D3	19.51	19.91	55.74	63.97	46.46	36.84	18.01	20.16	28.11
9	A3	B3	C2	D1	49.36	47.75	88.7	87.74	77.86	75.98	51.16	50.86	35.58

− represents level 1 for noise factors, +represents level 2 for noise factors.

**Table 3:** S/N ratio for each factor in each level.

Factor/level	[ 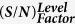 ]_j_			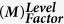
	j = 1	j = 2	j = 3	
A1	22.08	15.24	12.76	16.69
A2	24.09	27.81	23.75	25.22
A3	25.94	28.11	35.58	29.88
B1	22.08	24.09	25.94	24.04
B2	15.24	27.81	28.11	23.72
B3	12.76	23.75	35.58	24.03
C1	22.08	23.75	28.11	24.65
C2	15.24	24.09	35.58	24.97
C3	12.76	27.81	25.94	22.17
D1	22.08	27.81	35.58	28.49
D2	15.24	23.75	25.94	21.64
D3	12.76	24.09	28.11	21.66

**Table 4:** Contribution ratio of each factor.

Factor	DOF_F_	SS_F_	*ρ*%	SS_T_	V_E_
Dose; A	2	9749.564	29.582	31420.366	227.454
Treatment time; B	2	1679.934	3.899		
Concentration of modifier; C	2	875.011	1.337		
Particle diameter; D	2	4786.229	13.785		

**Table 5:** The average of removal efficiency results of a certain factor in the *k*th level ( 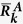 , 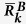 , 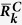 , 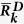 ) and the average of total removal efficiency ( 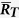 ).

Level	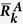	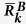	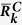	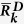	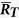
1	15.019	23.168	26.465	40.133	28.611
2	27.373	27.760	33.528	22.458	
3	43.442	34.907	25.841	23.243	

**Table 6:** Langmuir and Freundlich isotherm model parameter for Cr (VI) biosorption on SMPM.

Langmuir			Freundlich		
*q*_*m*_ (mg/g)	b (l/mg)	R^2^	*k*_*f*_ (mg/g)	n (l/mg)	R^2^
34.483	0.008324	0.9811	0.211	1.306	0.9991

**Table 7:** Thermodynamic parameters of Cr biosorption on SMPM.

Biosorbent	Δ*H* (kJ mol^−1^)	Δ*S* (kJ mol^−1^)	Δ*G*(kJ mol^−1^)		
			283.15 K	293.15 K	303.15 K
SMPM	17.532	0.0693	−1.94	−3.08	−3.33
